# The Late Dr. Alexander Nasmyth

**Published:** 1849-07

**Authors:** 


*The Late Dr. Alexander Nasmyth.*—We have recently received from our English correspondent, with other valuable matter, a brief obituary of the gentleman whose name heads this notice: but as we announced his death in the January number of the Journal, we shall omit its publication. We hope his biography will, ere long, be written by some one possessing the facts necessary to enable him to do justice to one who, during his brilliant professional career, contributed so eminently to the advancement of dental science. Such a work would be as gladly received by the members of the profession in this country as in England. The memory of Alexander Nasmyth will be as fondly and lastingly cherished here as there. For many years previously to his death, it was our good fortune to number him among our most highly prized correspondents, and we have little doubt, from our knowledge of his devotion to the advancement of his profession, that the illness which ultimately terminated his life, was brought on by severe and unwearied physical and mental toil. Long and deeply will his loss be mourned.—*Balt. Ed.*

